# Outbreak of *Pandoraea commovens* Infections among Non–Cystic Fibrosis Intensive Care Patients, Germany, 2019–2021

**DOI:** 10.3201/eid2911.230493

**Published:** 2023-11

**Authors:** Tassilo Kruis, Peter Menzel, Rolf Schwarzer, Solveigh Wiesener, Felix Schoenrath, Frank Klefisch, Miriam Stegemann, Frieder Pfäfflin

**Affiliations:** Labor Berlin Charité Vivantes GmbH, Berlin, Germany (T. Kruis, P. Menzel, R. Schwarzer);; German Heart Center Berlin, Berlin (S. Wiesener, F. Schoenrath); Paulinenkrankenhaus, Berlin (F. Klefisch);; Charité–Universitätsmedizin Berlin, Berlin (M. Stegemann, F. Pfäfflin)

**Keywords:** *Pandoraea commovens*, bacteria, outbreak, non–cystic fibrosis patients, antimicrobial resistance, respiratory infections, Germany

## Abstract

*Pandoraea* spp. are gram-negative, nonfermenting rods mainly known to infect patients with cystic fibrosis (CF). Outbreaks have been reported from several CF centers. We report a *Pandoraea* spp. outbreak comprising 24 non-CF patients at a large university hospital and a neighboring heart center in Germany during July 2019–December 2021. Common features in the patients were critical illness, invasive ventilation, antimicrobial pretreatment, and preceding surgery. Complicated and relapsing clinical courses were observed in cases with intraabdominal infections but not those with lower respiratory tract infections. Genomic analysis of 15 isolates identified *Pandoraea commovens* as the genetically most similar species and confirmed the clonality of the outbreak strain, designated *P. commovens* strain LB-19-202-79. The strain exhibited resistance to most antimicrobial drugs except ampicillin/sulbactam, imipenem, and trimethoprim/sulfamethoxazole. Our findings suggest *Pandoraea* spp. can spread among non-CF patients and underscore that clinicians and microbiologists should be vigilant in detecting and assessing unusual pathogens.

The widespread use of matrix-associated laser desorption/ionization time-of-flight (MALDI-TOF) mass spectrometry, 16s rRNA sequencing, and whole-genome sequencing (WGS) have improved diagnostic accuracy. However, in using those methods, microbiologists and clinicians can be confronted with uncommon gram-negative, nonfermenting bacteria. Those microorganisms originate from the environment, and their pathogenic potential is often unclear. When they are cultured from a clinical specimen, determining whether such a finding represents a true pathogen or a contaminant can be difficult.

One example is the genus *Pandoraea*, which was described in 2000 when researchers reclassified several *Burkholderia*- or *Ralstonia*-like organisms cultured from specimens of patients with cystic fibrosis (CF) (*1*). *Pandoraea* spp. are found in soil and water habitats, where the bacteria contribute to soil formation and cycling of elements necessary for plant growth. By 2022, at least 29 species had been identified within the genus, 19 of which were detected in clinical samples, most often from CF patients (*2*). Those species are *P. anapnoica*, *P. anhela*, *P. aquatica*, *P. apista*, *P. bronchicola*, *P. capi*, *P. captiosa*, *P. cepalis*, *P. commovens*, *P. communis*, *P. faecigallinarum*, *P. iniqua*, *P. morbifera*, *P. nosoerga*, *P. norimbergensis*, *P. pneumonica*, *P. pnomenusa*, *P. pulmonicola*, and *P. sputorum* (*2*).

*Pandoraea* spp. can trigger inflammatory responses and interleukin 6 and 8 elevation in cultures of lung epithelial cells and bacteria from some isolates are capable of crossing lung epithelial cell monolayers (*3*,*4*). In an in vivo model for killing *Galleria mellonella* larvae, virulence of some *Pandoraea* strains was comparable to that of *Burkholderia cenocepacia* (*3*,*4*). Various other virulence and resistance factors found in other pathogens also can be found in *Pandoraea* spp. *(**5*,*6**)*.

Knowledge on the clinical significance of *Pandoraea* spp. is based on case reports and case series. *Pandoraea* spp. can chronically colonize lungs of CF patients and evolve over time by sequential mutations, leading to an adaptation to the CF host niche (*7*–*10*). Worsening lung function in CF patients has been linked to *Pandoraea* spp. colonization, but because CF patients often carry multiple other relevant pathogens, causality between *Pandoraea* spp. colonization and clinical deterioration is not always clear (*9*,*11*,*12*). The potential of *Pandoraea* spp. to cause acute illnesses has been exemplified by bloodstream and other life-threatening infections in CF patients and patients who received solid organ transplantation (*7*,*13*–*15*). 

Single cases of *Pandoraea* spp. infections in patient populations other than those with CF or solid organ transplantation have been documented. Cases have occurred among persons without apparent immunodeficiency, causing illnesses such as nosocomial pneumonia, including infections associated with COVID-19, as well as localized hemodialysis catheter infections, prosthetic valve endocarditis, and skull base osteomyelitis (*16*–*22*). Nosocomial acquisition and antimicrobial pretreatment seem to be common features among affected patients (*16*–*22*).

*Pandoraea* outbreaks have been documented in CF centers in Denmark and France, each comprising 6 patients (*9*,*12*). One in-depth analysis described a large *P. apista* cluster affecting 18 CF patients serviced at the pediatric and adult CF centers in a city in Scotland and 1 other patient from south England (*6*). We report a *Pandoraea* spp. outbreak during July 2019–December 2021 at a large university hospital and the directly neighboring heart center in Berlin, Germany, involving 24 non-CF patients colonized or infected with a novel *P. commovens* strain.

## Methods

### Patient Data

We retrospectively extracted patient data from hospital records. Data included length of hospital stay, time to isolate *Pandoraea* spp., antimicrobial drug treatment, intensive care unit (ICU) admission, renal dialysis, solid organ transplantation, underlying conditions exemplified by the Charlson Comorbidity Index (CCI) scores, and patient outcome. We classified patients as either colonized or infected according to the judgment of 2 infectious disease consultants. Detection of *Pandoraea* spp. from otherwise sterile sites, such as blood, or from intraabdominal specimens was considered as infection. Culture from nonsterile sites (e.g., respiratory samples) was considered colonization if further assessment of antibiotic prescriptions, physicians’ notes, laboratory values, and radiology and pathology findings did not reveal evidence of infection. For the diagnosis of pneumonia, >1 of the following criteria had to be met: new or progressive infiltrate, new or worsening respiratory signs and symptoms, or rising inflammatory markers and assessment of pneumonia by the treating physician. For difficult cases, 2 clinicians discussed and then agreed on a classification for each case (Appendix).

### Microbiology

All microbiological investigations were performed by Labor Berlin–Charité Vivantes GmbH in accordance with German Quality Standards for the Microbiological Diagnosis of Infectious Diseases (https://www.dghm.org). To detect aerobic bacteria, we plated clinical specimens on conventional solid media, then incubated in ambient air and 5% CO_2_ enriched atmosphere at 37°C. We read plates after 24 h and 48 h incubation. We tested suspicious gram-negative microorganisms on oxidase and catalase activity and identified microorganisms by using the VITEK 2 System (bioMérieux, https://www.biomerieux.com), VITEK MALDI-TOF mass spectrometry (bioMérieux), or both. Per our clinical routine, we performed antimicrobial susceptibility testing of *Pandoraea* spp. by using the VITEK 2 AST GN-233 card, GN-248 card, or both. In addition, we subjected several isolates to further genomic analyses. For those isolates, we performed broth microdilution by using MICRONAUT-S test plates (MERLIN Diagnostika GmbH, https://www.merlin-diagnostika.de), and tested the following antimicrobial agents: piperacillin, piperacillin/tazobactam, temocillin, ceftazidime, cefepime, ceftolozane/tazobactam, ceftazidime/avibactam, meropenem, imipenem, ertapenem, aztreonam, aztreonam/avibactam, ciprofloxacin, levofloxacin, gentamicin, tobramycin, amikacin, trimethoprim/sulfamethoxazole (TMP/SMX), fosfomycin, colistin, minocycline, and tigecycline. We used MIC strips (Liofilchem, https://www.liofilchem.com) to solve discrepancies or to test antimicrobial agents that failed or were not included in VITEK 2 or MICRONAUT-S panels. Were interpreted results according to non–species-related pharmacokinetic/pharmacodynamic (PK/PD) breakpoints published by the European Committee on Antimicrobial Susceptibility Testing (*23*).

We also conducted environmental investigations for a point source of *P. commovens*. For environmental investigations, we probed respiratory tubes, nebulizers, suction catheters, washing gloves, toothbrushes, nutrition solutions, inhalation solutions, oral medications such as painkillers in solution, eye and nasal ointments, and eye drops.

### Genomic Sequencing and Analysis

For exact species identification and determination of clonality, we subjected 15 clinical outbreak isolates to WGS, by using either the Nextera Flex (Illumina, https://www.illumina.com) or QIASeq FX (QIAGEN, https://www.qiagen.com) library preparation kits, according to the manufacturers’ protocols. In brief, we extracted and enzymatically fragmented 10–100 ng of DNA by using DNeasy PowerSoil Pro Kit (QIAGEN). We added indexed adapters and amplified libraries in limited-cycle PCRs. After clean-up, we quantified, normalized, and pooled sequence-ready libraries before sequencing by using 2× 250 cycles paired-end sequencing on a MiSeq (Illumina).

To close the genome, we performed nanopore sequencing on genomic DNA from isolate LB-19-202-79 from our outbreak on GridION (Oxford Nanopore Technologies, https://nanoporetech.com) using an R9.4 flow cell (Oxford Nanopore). We prepared the sequencing library by using the SQK-LSK109 Ligation Sequencing Kit (Oxford Nanopore), according to the manufacturer’s protocol. We performed basecalling by using Guppy version 5.0.11 (Oxford Nanopore) on the SUP accuracy setting in GridION.

After adapter-trimming the Illumina sequencing reads by using fastp version 0.20.0 (*24*), we performed de novo genome assemblies by using SPAdes assembler version 3.15.5 (*25*). For the assembly of nanopore sequencing reads, we tried several protocols using ont-assembly-snake version 1.0 (P. Menzel, unpub. data, https://doi.org/10.20944/preprints202208.0191.v1) and eventually chose the protocol that showed the least differences with *P. commovens* strain LMG 31010 (National Center for Biotechnology Information [NCBI] RefSeq accession no. GCF_902459615.1). For that protocol, we used Filtong (https://github.com/rrwick/Filtlong) to quality-filter nanopore reads that passed the basecalling quality filter to the top 500 megabases and assembled reads using Flye version 2.9 (*26*). Then, we polished the initial assembly with the ONT reads by using Racon version 1.4.20 (*27*) and Medaka version 1.4.3 (https://github.com/nanoporetech/medaka) and polished the Illumina reads by using Polypolish version 0.5.0 (*28*). We rotated the final assembly to start at the *dnaA* gene.

We screened the genome assembly of LB-19-202-79 against all available *Pandoraea* spp. assemblies in the NCBI RefSeq database (*29*) as of July 21, 2022, by using mash-screen version 2.3 (*30*). We calculated average nucleotide identity (ANI) between genome assemblies by using FastANI version 1.33 (*31*). We used andi version 0.12 (*32*) to calculate pairwise genetic distances between the assembled genomes, from which we constructed a phylogenetic tree comprising the isolate assemblies and the closest *Pandoraea* spp. by using the neighbor joining method of the ape package version 5.6 (*33*).

We uploaded the genome assembly to GenBank for annotation by using the NCBI Prokaryotic Genome Annotation Pipeline (*34*). The uploaded assembly was then automatically annotated by the Bacterial and Viral Bioinformatics Resource Center (BV-BRC) pipeline (https://www.bv-brc.org).

### Ethics, Consent, and Permissions

The study was approved by the internal review board of Charité–Universitätmedizin Berlin (registry no. EA4/145/21). The need for informed consent was waived because the study was retrospective.

## Results

**Figure 1:**
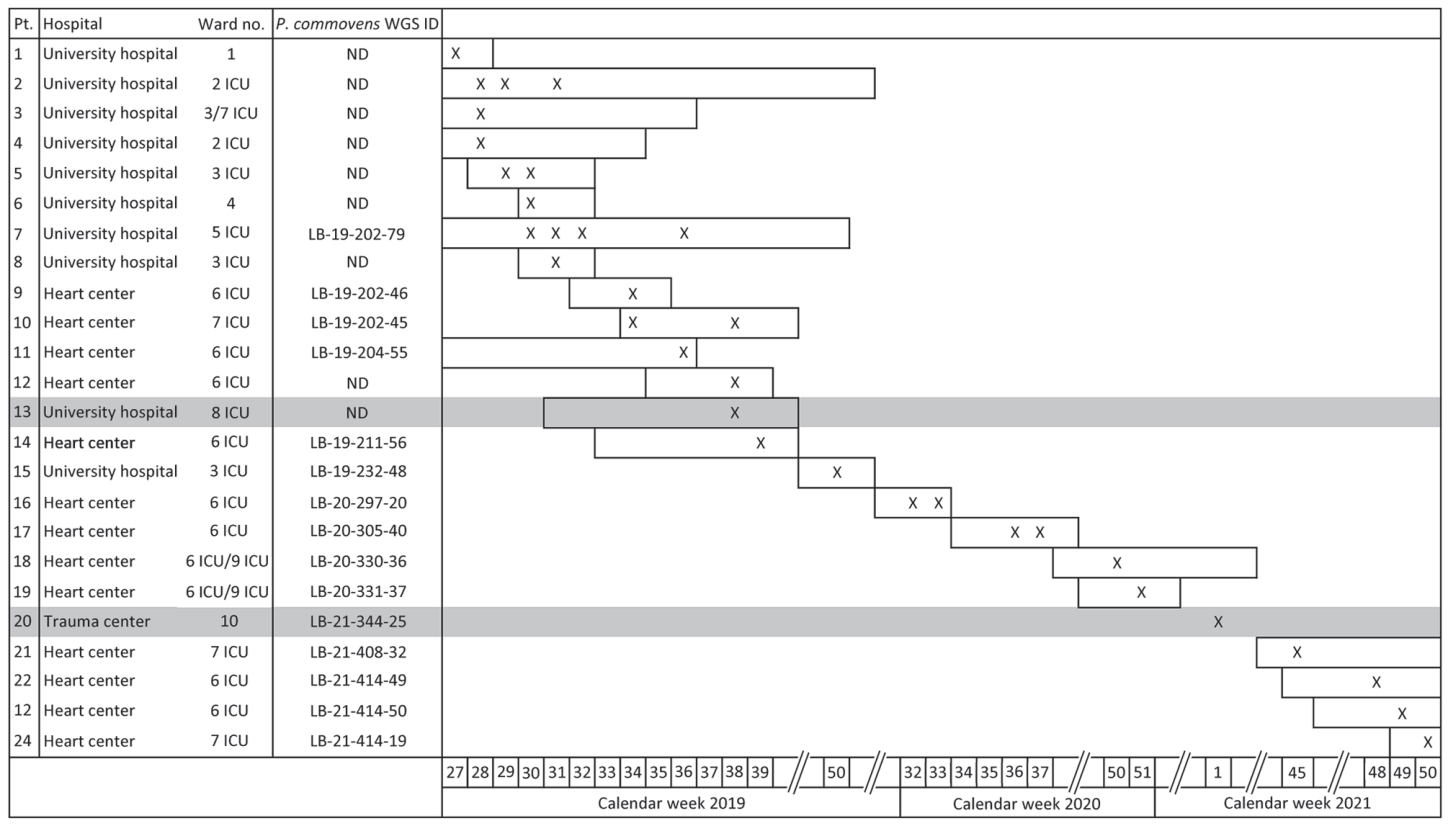
Timeline of an outbreak of *Pandoraea commovens* among non–cystic fibrosis intensive care patients, Germany, 2019–2021. The cases clustered at Charité Campus Virchow Klinikum (university hospital) and Deutsches Herzzentrum Berlin (heart center), 2 neighboring institutions that are on the same grounds and staff and patients regularly move between the 2 institutions. Wards 1–7 are located at the same grounds; wards 8 and 10 are located in facilities elsewhere in Berlin (indicated by grey background). X symbols indicate calendar weeks with detection of *P. commovens*.; horizontal bars in timelines indicate length of stay. ICU, intensive care unit; ND not done; Pt., patient; WGS ID, whole-genome sequencing identification number corresponding to the numbering in phylogenetic tree (Figure 2).

During July 2019–December 2021, we registered phenotypically identical *Pandoraea* spp. isolates in specimens from 24 patients, which is 8 times the number of all *Pandoraea* spp. detected at our laboratory in the 3 previous years (2016–2018). The cases clustered at Charité Campus Virchow Klinikum (CVK) and Deutsches Herzzentrum Berlin (DHZB), 2 neighboring institutions that are on the same grounds; staff and patients regularly move between the 2 institutions. Thirteen patients were treated at DHZB and 9 were treated at CVK. One other patient was treated at Charité Campus Benjamin Franklin and 1 at Unfallkrankenhaus Berlin, a major trauma center; both of those institutions are in different districts of the city. A total of 7 ICUs, 3 at DHZB and 4 at Charité CVK, and 3 regular wards were affected by the outbreak. Cases were first observed at Charité CVK, then the outbreak shifted after a patient was transferred to 2 ICUs at DHZB. Since late August 2019, nearly all isolates have been recovered on those 2 ICUs (Figure 1). Environmental investigations performed at those ICUs in September 2019 did not reveal any point source.

### Patient Information and Outcomes

Among the 24 patients whose cultures grew *Pandoraea* spp., the median age was 67 (range 45–81) years; 50% were male and 50% female. Clinical data were available on 23 patients. Median time from admission to *Pandoraea* spp. detection was 22 days; 22 (96%) of the 23 patients were treated in ICUs. Patients had numerous underlying conditions, and the median CCI was 6 (range 0–13). All patients had received antimicrobial drug treatment during their hospital stays before *Pandoraea* spp. detection, 65% had undergone surgery, and 65% received mechanical ventilation (Table 1).

**Table 1:** Patient characteristics in an outbreak of *Pandoraea commovens* among non–cystic fibrosis intensive care patients, Germany, 2019–2021*

Characteristics	Value†	IQR
Sex		
M	12 (50)	NA
F	12 (50)	NA
Age, y	67 (45–81)	61–69
Length of stay, d	51 (0–131)	32–105
Discharged alive	17 (74)	NA
Time to detect *P. commovens*, d	22 (0–131)	11–45
Intensive care unit admission	22 (96)	NA
Invasive ventilation	15 (65)	NA
Renal replacement therapy	7 (30)	NA
Surgery	15 (65)	NA
Antimicrobial drug therapy		
During hospitalization	23 (100)	NA
During previous 3 mo	14 (82)	NA
Immunosuppressed	5 (22)	NA
Charlson Comorbidity Index	6 (0–13)	3–6
Vascular disease or disorder		
Myocardial infarction	4 (17)	NA
Congestive heart failure	14 (61)	NA
Peripheral vascular disease	3 (13)	NA
Cerebrovascular accident	3 (13)	NA
Chronic lung disease	6 (26)	NA
Peptic ulcer disease	1 (4)	NA
Moderate to severe liver disease	3 (13)	NA
Moderate to severe CKD	9 (39)	NA
Diabetes mellitus	5 (22)	NA
Diabetes mellitus with end-organ damage	4 (17)	NA
Cancer		
Solid tumor	2 (9)	NA
Leukemia	2 (9)	NA
Lymphoma	2 (9)	NA

*Data reflect 23 cases, except for sex, median age, and median length of hospitalization (n = 24). CKD, chronic kidney disease. †Values are no. (%) or median (range).

We considered 12 patients colonized and 10 patients infected. For 2 cases, we were unable to make a classification. All 10 of the infected patients were on mechanical ventilation, compared with only 4 (33%) colonized patients. Among infected patients, 8 had lower respiratory tract infections and 2 had intraabdominal infections. In 5 infected patients, *Pandoraea* spp. was part of a polymicrobial culture, and in the other 5 infected patients, no other pathogens were detected. *Pandoraea* spp. infections were treated with imipenem in 8 patients and meropenem in 2 patients. TMP/SMX was administered as stepdown therapy in 1 patient after initial treatment with imipenem. All respiratory tract infections resolved, whereas the 2 patients with intraabdominal infections had complicated clinical courses that involved several surgical interventions and protracted administration of various antimicrobial agents in both cases (Appendix). 

Among the 10 infected patients, 4 died during hospitalization. None of those deaths were judged to be directly related to the *Pandoraea* spp. infections (Table 2).

**Table 2:** Clinical details for an outbreak of *Pandoraea commovens* among non–cystic fibrosis intensive care patients, Germany, 2019–2021.

Case no.	Age, y/sex	Course of infection	Underlying conditions	*Pandoraea*-positive specimens	Antimicrobial drug therapy
1	66/M	Colonization only	Chronic lung disease, solid tumor	Bile	None
2	67/F	Necrotizing pancreatitis with superinfection; *P. commovens* repeatedly detected during 4 weeks; needed mechanical ventilation, renal dialysis; and repeated endoscopic necrosectomies; discharged alive	Hyperparathyreoidism with hypercalcemia; diabetes mellitus with chronic kidney disease	Abdominal puncture ×2; wound swab; ascites puncture ×2	Meropenem
3	59/M	Colonization only	Double lung transplantation due to pulmonary hypertension; immunosuppression with mycophenolate-mofetil, cyclosporin, and corticosteroids	Wound swab	None
4	81/M	Colonization only	Congestive heart failure; status post myocardial infarction; diabetes mellitus with chronic kidney disease; moderate to severe hepatopathy	Bronchial lavage; tracheobronchial secretion ×2	None
5	77/F	Colonization only	Congestive heart failure; myocardial infarction; peripheral vascular disease	Bronchial lavage; tracheobronchial secretion	None
6	68/F	Colonization only	Vertebral fracture; coronary heart disease	Throat swab	None
7	45/M	Pancreatitis after endoscopic retrograde cholangiography, acute respiratory distress syndrome, complicated intraabdominal infection with bacteremia; *P. commovens* repeatedly detected during 3 weeks; multiple surgeries for source control; renal dialysis; died, but death not attributable to *P. commovens* infection	Gallstones	Blood culture; intraabdominal swab ×5; intraabdominal biopsy; tracheobronchial secretion ×2	Imipenem, TMP/SXT
8	71/F	Colonization only	Status post cardiogenic and septic shock with multiorgan failure; diabetes mellitus with chronic kidney disease; moderate to severe hepatopathy	Bronchial lavage	None
9	69/M	Ventilator-associated pneumonia after mitral valve replacement and coronary artery bypass graft; discharged alive	Congestive heart failure	Tracheobronchial secretion	Imipenem
10	69/M	Colonization only	Cardiomyopathy with left and right ventricular assist device; peripheral vascular disease; lymphoma	Throat swab ×2	None
11	49/F	Unclear; died on same day as *P. commovens* sampling	Cardiomyopathy, evaluated for heart transplantation	Tracheobronchial secretion	None
12	75/F	Colonization only	Thoracic endovascular aortic repair due to aneurysm; chronic kidney disease	Throat swab	None
13	59/F	Ventilator-associated pneumonia; discharged alive	Central nervous system diffuse large B cell lymphoma; neutropenic sepsis after methotrexate, cytarabine, thiotepa, and rituximab	Tracheobronchial secretion	Meropenem
14	60/M	Ventilator-associated pneumonia; discharged alive	Congestive heart failure; cerebrovascular disease; peptic ulcer disease; solid tumor	Tracheobronchial secretion	Imipenem
15	67/F	Septic shock, ventilator-associated pneumonia, renal dialysis; died, but likely not attributable to *P. commovens* infection	Secondary acute myeloid leukemia, status post allogeneic stem cell transplantation; immunosuppression with cyclosporine A; right heart failure; pulmonary hypertension	Tracheobronchial secretion	Imipenem
16	62/M	Ventilator-associated pneumonia; discharged alive	Congestive heart failure, status post myocardial infarction; chronic lung disease	Tracheobronchial secretion ×3	Imipenem
17	75/F	Ventilator-associated pneumonia; died, but likely not attributable to *P. commovens* infection	Congestive heart failure; chronic kidney disease	Tracheobronchial secretion; throat swab	Imipenem
18	69/F	Colonization only	Congestive heart failure; cerebrovascular disease; solid tumor	Tracheobronchial secretion	None
19	67/M	Colonization only	Congestive heart failure; chronic lung disease; diabetes mellitus with chronic kidney disease; moderate to severe hepatopathy	Tracheobronchial secretion	None
20	78/F	ND	ND	Throat swab	ND
21	63/F	Colonization only	Congestive heart failure; chronic lung disease; chronic kidney disease	Tracheobronchial secretion	None
22	62/M	Ventilator-associated pneumonia after myocardial infarction; discharged alive	Congestive heart failure; peripheral artery and cerebrovascular disease; chronic lung disease; chronic kidney disease	Tracheobronchial secretion	Imipenem
23	63/M	Colonization only	Congestive heart failure; stroke related to atrial fibrillation; chronic kidney disease	Tracheobronchial secretion	ND
24	55/M	Hospital-acquired pneumonia, mediastinitis, neutropenic sepsis; died, but likely not attributable to *P. commovens* infection	Infected aortic prosthesis; secondary acute myeloid leukemia; chronic myelomonocytic leukemia	Sputum	Imipenem

*Whole-genome sequencing was performed on isolates from patients 7, 9–11, and 14–24. However, due to the local and temporal relationships, identical colony morphology, and antibiotic susceptibilities, we assumed that the correct species was *P. commovens* in all patients reported here. ND, no data; TMP/SXT, trimethoprim/sulfamethoxazole.

### Microbiology Results

During July 2019–December 2021, *Pandoraea* spp. was cultured from 43 clinical specimens, including throat swabs, respiratory secretions, bile, ascites, intraabdominal specimens, and wound swabs. Numerous blood cultures were collected from 19 of the 24 patients. However, *Pandoraea* spp. was only detected in 1 blood culture from a patient with a complicated intraabdominal infection. *Pandoraea* spp. isolates grew readily after overnight culture on commercial solid media, such as Columbia blood or MacConkey agars. Single colonies appeared pale to grayish, displayed weak oxidase activity, and were catalase negative. Neither a mucoid phenotype nor small colony variants were observed.

Using the VITEK 2 GN ID card, identification was possible only to the genus level. In 4 of 17 tested isolates, a reliable discrimination between *Pandoraea* spp. and *Bordetella hinzii* could not be made by VITEK 2. In the remaining 13 isolates, we identified *Pandoraea* spp. with probabilities ranging from 95% to 99%. All 36 isolates tested by VITEK MALDI-TOF mass spectrometry were identified as *P. sputorum* with a score of 99.9. We were able to perform WGS to differentiate *P. commovens* from *P. sputorum* on isolates from patients 7, 9–11, and 14–24, as described in the next section. Because of the local and temporal relationship, identical colony morphology and antimicrobial susceptibilities, we assumed that the correct species identification was *P. commovens* in all patients reported in this outbreak and that *P. sputorum* was a misidentification resulting from limitations in the VITEK mass spectrometry database.

We performed susceptibility testing on 35 isolates. For susceptibility testing methods, agreement between VITEK 2 and MICRONAUT-S broth microdilution plates was good for most tested antimicrobial agents. According to EUCAST PK/PD breakpoints, MICs obtained for ampicillin/sulbactam and imipenem were consistently found to be susceptible at standard dosing levels. MICs for TMP/SMX were <20 mg/L in all tested isolates. Cephalosporins including ceftazidime/avibactam, ceftolozane/tazobactam, and cefiderocol tested resistant, as did fluoroquinolones, aminoglycosides, tetracyclines, and colistin (Table 3).

**Table 3:** Antimicrobial susceptibilities of *Pandoraea commovens* isolates in an outbreak among non–cystic fibrosis intensive care patients, Germany, 2019–2021

Antimicrobial drugs tested	MIC, mg/L	Breakpoints, mg/L	
		Susceptible	Resistant
Ampicillin	>32	<2	>8
Ampicillin/sulbactam	<2	<2	>8
Piperacillin	8 to >128	<8	>16
Piperacillin/tazobactam	<1 to >128	<8	>16
Temocillin	>128	IE	IE
Cefotaxime	8–16	<1	>2
Ceftazidime	>64	<4	>8
Cefepime	>64	<4	>8
Ceftolozane/tazobactam	16 to >256	<4	>4
Ceftazidime/avibactam	16 to >256	<4	>8
Cefiderocol	>256	<2	>2
Meropenem	1–8	<2	>8
Imipenem	<0.25 to 1	<2	>4
Imipenem/relebactam	<1	<2	>2
Ertapenem	<0.5 to >8	<0.5	>0.5
Aztreonam	32 to >128	<4	>8
Aztreonam/avibactam	>128	NA	NA
Ciprofloxacin	>4	<0.25	>0.5
Moxifloxacin	>8	<0.25	>0.25
Gentamicin	8 to >16	<0.5	>0.5
Tobramycin	>16	<0.5	>0.5
Amikacin	>64	1	>1
TMP/SXT	<20	IE	IE
Fosfomycin	128 to >256	IE	IE
Colistin	>16	IE	IE
Minocycline	1–4	IE	IE
Tigecycline	1	<0.5	>0.5
Doxycycline	>16	IE	IE

*MIC results are from susceptibility testing of 35 *Pandoraea* spp. isolates. The table comprises results of different test methods VITEK 2 (bioMérieux, https://www.biomerieux.com), MICRONAUT-S broth microdilution (MERLIN Diagnostika GmbH, https://www.merlin-diagnostika.de), and MIC strips (Liofilchem, https://www.liofilchem.com). IE, insufficient evidence, no breakpoints available; NA, not applicable; TMP/SXT, trimethoprim/sulfamethoxazole. †According to pharmacokinetic/pharmacodynamic (non–species related breakpoints from European Committee on Antimicrobial Susceptibility Testing Clinical Breakpoint Tables version 11.0, 2021 (http://www.eucast.org).

Discrepancies between VITEK 2 and microdilution were apparent for piperacillin and piperacillin/tazobactam; higher MICs were detected using VITEK 2 (range 16 to >128 mg/L for both) than with microdilution (range <4 to 32 mg/L for piperacillin and <1 to 2 mg/L for piperacillin-tazobactam). MICs for meropenem and ertapenem were also higher in VITEK 2 (range 4–8 mg/L and 1 to >8 mg/L, respectively) than in microdilution (range 1–4 mg/L and <0.5 to 1 mg/L, respectively).

### Genomic Characterization and Phylogeny

**Figure 2:**
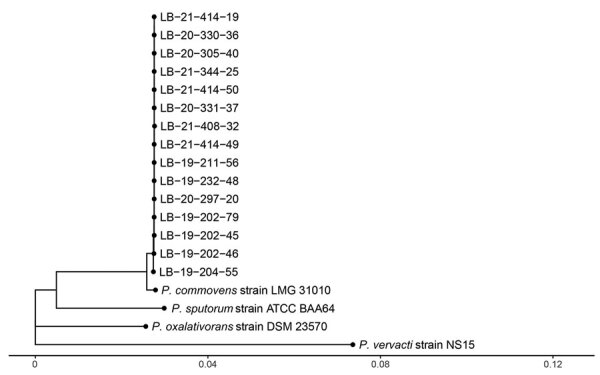
Phylogenetic tree of isolates from an outbreak of *Pandoraea commovens* among non–cystic fibrosis intensive care patients, Germany, 2019–2021. Genome assemblies from 15 isolates (labeled LB) compared with *Pandoraea* spp. genomes in the National Center for Biotechnology Information RefSeq database (https://www.ncbi.nlm.nih.gov) found the genome assembly GCF_902459615.1 of *P. commovens* strain LMG 31010 was the most similar. The tree was created by using neighbor joining the calculated pairwise phylogenetic distances between genome assemblies and available database sequences. Scale bar indicates nucleotide substitutions per site.

We screened the genome assemblies from 15 isolates from our outbreak against genomes in RefSeq. We found the genome assembly GCF_902459615.1 of *P. commovens* strain LMG 31010 to be the most similar, then *P. sputorum* strain ATCCBAA64, and *P. oxalativorans* strain DSM 23570. The average genome-wide nucleotide identity between isolate LB-19-202-79 and GCF_902459615.1 was 99.5% and identity between LB-19-202-79 and *P. sputorum* GCF_900187205.1 was 94.1%. Thus, we designated our strain as *P. commovens* strain LB-19-202-79. The phylogenetic tree derived from the pairwise phylogenetic distances showed that all our isolates are closely related to each other and distinct from *P. commovens* strain LMG 31010 and other *Pandoraea* spp. (Figure 2). We concluded that the outbreak isolates were from a single origin.

The assembly of isolate LB-19-202-79 (NCBI BioSample no. SAMN30015177) from nanopore sequencing data yielded 1 circular chromosome of 5.9-megabase length (GC content 57%; GenBank accession no. CP102780) and 1 plasmid of 80.7 kb length (GC content 63%, 2 copies; GenBank accession no. CP102779).

A search of the plasmid sequence against the NCBI BLAST nucleotide database (https://blast.ncbi.nlm.nih.gov) revealed several alignments to the plasmid of *Burkholderia aenigmatica* strain CMCC(B)23010 (GenBank accession no. CP091649.1), totaling ≈22 kb and ≈99.9% sequence identity. That strain is a member of the *Burkholderia cepacia* complex and was originally isolated from water purified for pharmaceuticals. Apart from that, we only found alignments to transposase genes in *Klebsiella pneumoniae* plasmids.

The annotation of the LB-19-202-79 chromosome yielded 2 β-lactamase family proteins that are also found in the *P. commovens* strain LMG 31010 genome assembly and have >99% amino acid identity. The β-lactamase with locus tag NTU39_20675 was identified as an oxacillinase (OXA) 62 family carbapenem-hydrolyzing class D enzyme and now is denoted as allele *bla*OXA-1149 in the NCBI Reference Gene Catalog. The β-lactamase with locus tag NTU39_00730 was identified as a class C β-lactamase. We analyzed the complete resistome comprising all genes associated with antimicrobial resistance as determined by the BV-BRC genome annotation (Appendix Table). The complete genome annotation is available at BV-BRC (accession no. 2508289.5; https://www.bv-brc.org/view/Genome/2508289.5).

## Discussion

We describe a large *Pandoraea* spp. outbreak comprising 24 non-CF patients. In contrast to earlier outbreaks that took place uniformly among CF patients (*6*,*9*,*12*), none of the patients in this outbreak had CF. However, all but 1 patient were treated in an ICU immediately before or during the time when *P. commovens* was isolated. All patients had received antimicrobial drugs before *P. commovens* isolation, and all likely acquired the pathogen in the hospital. Most of the patients had undergone surgery or were on mechanical ventilation, and their overall CCI was high (median 6, range 0–13). Those observations align with earlier case reports on *Pandoraea* spp. infections among non-CF patients (*16*–*21*).

As described by others (*35*), we experienced difficulties in correctly identifying the species of the outbreak strain. In several cases, *P. commovens* was misidentified as *B. hinzii* by biochemical means. Because *P. commovens* was not identified as a separate species before November 2019 (*2*), VITEK MALDI-TOF mass spectrometry analysis misidentified the outbreak strain as *P. sputorum*.

*Pandoraea* spp. can harbor multiple antimicrobial resistance and biodegradation genes, enabling the pathogen to persist in the hospital environment (*6*). Our resistome analyses and the course of this outbreak that lasted for 2.5 years suggest that *P. commovens* LB-19-202-79 is equipped with such an armament.

*Pandoraea* spp. exhibit resistance to most antimicrobial agents, including penicillins, cephalosporins, fluoroquinolones, aminoglycosides, and colistin, but frequently are susceptible to imipenem and TMP/SMX (*14*,*36*,*37*). Resistance is mediated by different efflux pumps and β-lactamases with a carbapenem-resistant phenotype observed in isolates carrying OXA-62 or a homologue to OXA-153, both carbapenem-hydrolyzing oxacillinases. OXA-62 hydrolyzes meropenem more efficiently than imipenem, but expanded-spectrum cephalosporins are only poor substrates (*5*,*6*,*38*). Among the β-lactamase family proteins detected in our strain, one was identified as an OXA-62 family carbapenem-hydrolyzing class D β-lactamase, now denoted as OXA-1149. However, all our *P. commovens* isolates were susceptible to imipenem but showed elevated MICs (1–8 mg/L) for meropenem, corresponding to susceptible to increased exposure according to EUCAST PK/PD non–species related breakpoints. The high-level resistance of *P. commovens* to all cephalosporins might at least in part be mediated by the expression of a class C β-lactamase (*39*). Although all 8 patients with respiratory tract infections recovered with a single course of antimicrobial drugs, the 2 patients with *P. commovens* intraabdominal infections had relapsing courses of disease. *Pandoraea* spp. are environmental bacteria and can thrive in wet settings, such as an abdominal area undergoing multiple surgeries. Under such circumstances, armed with a class D carbapenemase, *P. commovens* LB-19-202-79 might withstand even prolonged targeted antimicrobial treatment, as noted in patient 7 (Appendix).

The assessment of the clinical significance of detection of *P. commovens* in the patients in this outbreak was not always straightforward, especially in cases where *Pandoraea* spp. was cultured from respiratory secretions. Some cases could easily be classified as colonization, such as when only throat swab samples were positive and patients had no other signs and symptoms of infection. However, *P. commovens* was part of polymicrobial cultures in some patients, and its role in those cases was difficult to estimate. *P. commovens* was the only relevant pathogen that could be detected in 5 of 8 patients with nosocomial pneumonia; however, it was only detected in low to intermediate quantities. Those cases were classified as infections because patients had nosocomial pneumonia, which cleared after administration of targeted antimicrobial drug treatment for *P. commovens*. The pathogenicity of *P. commovens* in the 2 patients with complicated intraabdominal infections seemed evident. *P. commovens* was the predominant pathogen and was repeatedly isolated from different materials, even from blood culture in 1 case.

The first limitation of this study is that we were not able to sequence all *Pandoraea* spp. isolates from the outbreak. Some uncertainty remains about whether all isolates belonged to the outbreak strain *P. commovens* LB-19-202-79. However, given the identical phenotypic features and the temporal and spatial relationship, we assume the same strain was responsible for all cases. Second, we were not able to find an environmental source. Third, detection of *Pandoraea* spp. could not easily be classified as colonization or infection in several patients; however, that is a well-known dilemma when low-virulent pathogens are cultured from nonsterile sites, such as the respiratory tract.

In conclusion, our sequence analysis highlights the advantage of bacterial WGS for exact species identification and typing of outbreak isolates. On the basis of these findings, we conclude that *Pandoraea* spp. are not only capable of spreading among CF patients, as described before, but also to non-CF patients. The bacteria can also cause outbreaks on ICUs, in particular affecting patients with a history of intensive antimicrobial pretreatment, multiple abdominal surgeries, and mechanical ventilation. This outbreak report underscores the critical role of vigilance among both clinicians and microbiologists when unusual pathogens occur and the need for access to modern molecular sequencing techniques in hospital laboratories.

Appendix Additional information on an outbreak of *Pandoraea commovens* among non–cystic fibrosis intensive care patients, Germany, 2019–2021.
