# Supplementary material for: Outbreak of Pandoraea commovens Infections among Non–Cystic Fibrosis Intensive Care Patients, Germany, 2019–2021
Source: Emerg Infect Dis. 2023 Nov;29(11):2229–37. doi: 10.3201/eid2911.230493 (PMC10617358; doi:10.3201/eid2911.230493)
Supplement: Appendix — Additional information on an outbreak of Pandoraea commovens among non–cystic fibrosis intensive care patients, Germany, 2019–2021. [file 23-0493-Techapp-s1.pdf]

Article DOI: https://doi.org/10.3201/eid2911.230493

EID cannot ensure accessibility for supplementary materials supplied by authors. Readers who have difficulty accessing supplementary content should contact the authors for assistance.

Outbreak of *Pandoraea commovens* among Non–Cystic Fibrosis Intensive Care Patients, Germany, 2019–2021e

Appendix

Case vignettes

Examples of Difficult Patient Classifications as Infection or Colonization

Patient 4 was an 85-year-old man with coronary heart disease, congestive heart failure, diabetes mellitus, chronic kidney disease, and liver cirrhosis. He was admitted with non–ST-elevation myocardial infarction and acute kidney injury requiring renal replacement therapy. He remained in intensive care for several weeks with several severe complications (e.g., *Candida* spp. blood stream infection, ventilator-associated pneumonia due to *Pseudomonas aeruginosa*, *Clostridioides difficile* infection). Numerous specimens were sent for microbiological investigation. Two respiratory samples 2 days apart grew *P. commovens*. At the time of identification, the patient was mechanically ventilated but did not receive antibiotics apart from caspofungin for *Candida* spp. blood stream infection. Radiology revealed pleural effusions and lung infiltrates, which were both in regression. Three days after the last detection of *P. commovens*, *P. aeruginosa* was cultured from tracheal secretions. The patient's clinical status deteriorated and meropenem was started. This case was classified as colonization.

Patient 14 was a 60-year-old woman who had undergone hematopoietic stem cell transplantation for acute myeloid leukemia. She was admitted to intensive care with right heart failure due to pulmonary hypertension and acute kidney injury requiring renal replacement therapy. The course in the intensive care unit was complicated by reactivation of cytomegalovirus, pulmonary embolism, and several episodes of nosocomial pneumonia.

Numerous specimens were sent for microbiological investigation. *P. commovens* was cultured from tracheal secretions only once. At the time of identification, imaging of the lung revealed new infiltrates. Concomitantly, values of C-reactive protein rose to 136 mg/l. A diagnosis of nosocomial pneumonia was made and treatment with imipenem was initiated. Values of C-reactive protein declined thereafter, and the patient's clinical condition improved. This case was classified as infection.

Patients with Complicated Intraabdominal Infections

Patient 2 was admitted from an ICU of an external hospital with exudative pancreatitis due to hypercalcemia secondary to primary hyperparathyroidism. On admission, she was in septic shock necessitating fluid resuscitation, high-dose vasopressors, and antimicrobials. Oxygenation was severely impaired with a PaO_2/FIO_2 ratio of 80 mm Hg. Renal failure was addressed with renal replacement therapy. Hyperparathyroidism was treated with cinacalcet and intravenous bisphosphonates. Pancreatic fluid collections were drained. The patient's status stabilized, and she could be transferred to a normal ward. However, the course of the disease remained complicated. She had to be readmitted to the ICU with hypernatremia, intra-abdominal bleeding episodes, and superinfection of the pancreatic fluid collections. The latter required repeated endoscopic necrosectomies. Punctures from the abdomen and the ascites revealed *C. glabrata*, *E. faecium*, *Pandoraea* spp., *S. maltophilia*, and *S. epidermidis*. The patient received several courses of intravenous antimicrobials. *Pandoraea* was regarded as relevant as it was repeatedly cultured from normally sterile sites. A 4-week course of meropenem was administered, accompanied by vancomycin and caspofungin to address gram-positive bacteria and fungi, respectively. Thereafter, *Pandoraea* spp. could not be cultivated anymore from samples sent to the microbiology department. Surgery was eventually performed with resection of the pancreas tail, splenectomy, necrosectomy, and partial gastric resection. Furthermore, an adenoma of the parathyroid was removed. The patient's condition gradually improved following surgery. However, she had severe critical illness myopathy and peripheral neuropathy. After a total of almost 7 months, she could be transferred to rehabilitation.

Patient 7 was also admitted from the ICU of an external hospital. He had previously been healthy apart from a posttraumatic stress disorder and symptomatic gallstones. He developed exudative pancreatitis as a complication after endoscopic retrograde cholangiography (ERC) for the treatment of the gallstones. Initial treatment in the external hospital comprised intravenous

antibiotics, fluids, and analgesics. However, acute respiratory distress syndrome (ARDS) developed, and the patient was transferred to Charité - CVK for further treatment. The treatment in Charité - CVK was long and complicated. Open laparotomies had to be performed on several occasions for necrosectomies, adhesiolyses, abdominal lavage and reconstruction of the abdominal wall. Eventually, subtotal left-sided pancreatectomy, splenectomy, and subtotal colectomy had to be carried out with terminal ileostomy. *Pandoraea commovens* was first isolated from blood culture on day 74 after transfer with exact species identification possible only by whole-genome sequencing. In the following 18 days, *Pandoraea* spp. was also repeatedly isolated from intra-abdominal specimes (together with *C. albicans*, vancomycin-resistant enterococci, methicillin-resistant *S. aureus*, *S. epidermidis*, and *E. cloacae*) and from tracheobronchial secretions (together with *E. cloacae* and *C. albicans*). Susceptibility testing revealed low MICs to imipenem and TMP-SMX. Thus, imipenem was administered for a total of 33 days accompanied by antimicrobials addressing gram-positive bacteria and fungi. TMP-SMX was added after 30 days of imipenem treatment at a dose of 800/160 mg q8h. Two days after stopping imipenem, *P. commovens* was again isolated within a polymicrobial culture from the abdominal cavity. Dosing of TMP-SMX was adjusted to 1600/320 mg q12 and TMP-SMX was given for a total of 30 days. Thereafter, *Pandoraea* spp. could no longer be detected from any specimen during the following 73 days on the ICU. The patient's status remained stable for roughly 2 months and then suddenly deteriorated. Imaging revealed gastric and duodenal perforation and leakage from the remainder of the rectosigmoid. ERC showed perforation of the bile duct. Surgery was deemed impossible. The patient eventually died from multiorgan failure.

Appendix Table. Antimicrobial resistance *Pandoraea commovens*

BRC ID	RefSeq locus tag	Gene	Product	Antimicrobial drugs
fig|2508289.5.peg.4200	NTU39_20675	*OXA-62*	Class D β-lactamase (EC 3.5.2.6) = >OXA-62 family, carbapenem-hydrolyzing	
fig|2508289.5.peg.5109	NTU39_25115	*rpsJ*	SSU ribosomal protein S10p (S20e)	
fig|2508289.5.peg.2427	NTU39_12160	*dxr*	1-deoxy-D-xylulose 5-phosphate reductoisomerase (EC 1.1.1.267)	Fosmidomycin
fig|2508289.5.peg.5396	NTU39_26555	*gidB*	16S rRNA (guanine(527)-N(7))-methyltransferase (EC 2.1.1.170)	Streptomycin
fig|2508289.5.peg.3124	NTU39_15500	*fabG*	3-hydroxyacyl-CoA dehydrogenase (EC 1.1.1.35), FabG4	Triclosan
fig|2508289.5.peg.4543	NTU39_22325	*kasA*	3-oxoacyl-[acyl-carrier-protein] synthase, KASII (EC 2.3.1.179)	Isoniazid, triclosan
fig|2508289.5.peg.2402	NTU39_12040	*alr*	Alanine racemase (EC 5.1.1.1)	D-cycloserine

BRC ID	RefSeq locus tag	Gene	Product	Antimicrobial drugs
fig|2508289.5.peg.4099	NTU39_20160	*AAC(6')-Ic,f,g,h,j,k,l,r–z*	Aminoglycoside N(6')-acetyltransferase (EC 2.3.1.82) = >AAC(6')-Ic,f,g,h,j,k,l,r-z	Tobramycin, kanamycin A, amikacin, dibekacin, sisomicin, gentamicin B, isepamicin, arbekacin, netilmicin, neomycin
fig|2508289.5.peg.4524	NTU39_22235	*pgsA*	CDP-diacylglycerol–glycerol-3-phosphate 3-phosphatidyltransferase (EC 2.7.8.5)	Daptomycin
fig|2508289.5.peg.68	NTU39_00730		Class C β-lactamase (EC 3.5.2.6)	
fig|2508289.5.peg.4200	NTU39_20675	*OXA-62* family	Class D β-lactamase (EC 3.5.2.6) ≥OXA-62 family, carbapenem-hydrolyzing	Oxacillin
fig|2508289.5.peg.4869	NTU39_23905	*Ddl*	D-alanine–D-alanine ligase (EC 6.3.2.4)	D-cycloserine
fig|2508289.5.peg.1404	NTU39_07215	*gyrA*	DNA gyrase subunit A (EC 5.99.1.3)	Clofazimine, ciprofloxacin, gatifloxacin, levofloxacin, moxifloxacin, nalidixic acid, ofloxacin, sparfloxacin, trovafloxacin
fig|2508289.5.peg.3	NTU39_00420	*gyrB*	DNA gyrase subunit B (EC 5.99.1.3)	Clofazimine, gatifloxacin, ciprofloxacin, levofloxacin, moxifloxacin, nalidixic acid, ofloxacin, sparfloxacin, novobiocin, coumermycin A1, clorobiocin, coumermycin, trovafloxacin
fig|2508289.5.peg.2263	NTU39_11380	*H-NS*	DNA binding protein H-NS	Cloxacillin, oxacillin, ciprofloxacin, norfloxacin, erythromycin, tetracycline
fig|2508289.5.peg.5138	NTU39_25260	*rpoB*	DNA-directed RNA polymerase β subunit (EC 2.7.7.6)	Rifamycin, daptomycin, rifabutin, rifampin
fig|2508289.5.peg.5137	NTU39_25255	*rpoC*	DNA-directed RNA polymerase β' subunit (EC 2.7.7.6)	Daptomycin
fig|2508289.5.peg.1925	NTU39_09720	*folA, Dfr*	Dihydrofolate reductase (EC 1.5.1.3)	Trimethoprim, brodimoprim, tetroxoprim, iclaprim
fig|2508289.5.peg.3664	NTU39_18075	*folP*	Dihydropteroate synthase (EC 2.5.1.15)	Sulfadiazine, sulfadimidine, sulfadoxine, sulfamethoxazole, sulfisoxazole, sulfacetamide, mafenide, sulfasalazine sulfamethizole, dapsone
fig|2508289.5.peg.3962	NTU39_19515	*fabV*	Enoyl-[acyl-carrier-protein] reductase [NADH] (EC 1.3.1.9), FabV ≥refractory to triclosan	Triclosan
fig|2508289.5.peg.1824	NTU39_09235	*GdpD*	Glycerophosphoryl diester phosphodiesterase (EC 3.1.4.46)	Daptomycin

BRC ID	RefSeq locus tag	Gene	Product	Antimicrobial drugs
fig|2508289.5.peg.4177	NTU39_20570	*GdpD*	Glycerophosphoryl diester phosphodiesterase (EC 3.1.4.46)	Daptomycin
fig|2508289.5.peg.986	NTU39_05160	*OxyR*	Hydrogen peroxide-inducible genes activator ≥OxyR	Isoniazid
fig|2508289.5.peg.4272	NTU39_21040	*Iso-tRNA*	Isoleucyl-tRNA synthetase (EC 6.1.1.5)	Mupirocin (pseudomonic acid)
fig|2508289.5.peg.4247	NTU39_20915	*MacB*	Macrolide export ATP binding/permease protein MacB	Erythromycin
fig|2508289.5.peg.1581	NTU39_08085	*MacB*	Macrolide export ATP binding/permease protein MacB	Erythromycin
fig|2508289.5.peg.1580	NTU39_08080	*MacA*	Macrolide-specific efflux protein MacA	Erythromycin
fig|2508289.5.peg.4246	NTU39_20910	*MacA*	Macrolide-specific efflux protein MacA	Erythromycin
fig|2508289.5.peg.2144	NTU39_10780	*MdfA/Cmr*	Multidrug efflux pump MdfA/Cmr (of MFS type), broad spectrum	Tetracycline, rhodamine, benzalkonium chloride
fig|2508289.5.peg.2326	NTU39_11700	*EmrAB-TolC*	Multidrug efflux system EmrAB-OMF, inner-membrane proton/drug antiporter EmrB (MFS type)	Nalidixic acid
fig|2508289.5.peg.5450	NTU39_26805	*EmrAB-TolC*	Multidrug efflux system EmrAB-OMF, inner-membrane proton/drug antiporter EmrB (MFS type)	Nalidixic acid
fig|2508289.5.peg.2327	NTU39_11705	*EmrAB-TolC*	Multidrug efflux system EmrAB-OMF, membrane fusion component EmrA	Nalidixic acid
fig|2508289.5.peg.4211	NTU39_20735	*MdtABC-TolC*	Multidrug efflux system MdtABC-TolC, inner-membrane proton/drug antiporter MdtB (RND type)	Novobiocin
fig|2508289.5.peg.2601	NTU39_13000	*MdtABC-TolC*	Multidrug efflux system MdtABC-TolC, inner-membrane proton/drug antiporter MdtB (RND type)	Novobiocin
fig|2508289.5.peg.2600	NTU39_12995	*MdtABC-TolC*	Multidrug efflux system MdtABC-TolC, inner-membrane proton/drug antiporter MdtC (RND type)	Novobiocin
fig|2508289.5.peg.4210	NTU39_20730	*MdtABC-TolC*	Multidrug efflux system MdtABC-TolC, inner-membrane proton/drug antiporter MdtC (RND type)	Novobiocin
fig|2508289.5.peg.2602	NTU39_13005	*MdtABC-TolC*	Multidrug efflux system MdtABC-TolC, membrane fusion component MdtA	Novobiocin
fig|2508289.5.peg.4212	NTU39_20740	*MdtABC-TolC*	Multidrug efflux system MdtABC-TolC, membrane fusion component MdtA	Novobiocin
fig|2508289.5.peg.4135	NTU39_20370	*MexXY-OMP*	Multidrug efflux system, membrane fusion component ≥MexX of MexXY/AxyXY	Acriflavin, amikacin, arbekacin, chloramphenicol, cefepime, ciprofloxacin, erythromycin, gentamicin C, meropenem, norfloxacin, ofloxacin, tetracycline, tobramycin
fig|2508289.5.peg.4200	NTU39_20675	*blaOXA*	Class D β-lactamase (EC 3.5.2.6) ≥OXA-62 family, carbapenem-hydrolyzing	
fig|2508289.5.peg.2328	NTU39_11710	*EmrAB-OMF*	Outer membrane factor (OMF) lipoprotein associated wth EmrAB-OMF efflux system	Nalidixic acid
fig|2508289.5.peg.4209	NTU39_20725	*MdtABC-OMF*	Outer membrane factor (OMF) lipoprotein associated wth MdtABC efflux system	Novobiocin
fig|2508289.5.peg.5109	NTU39_25115	*S10p*	SSU ribosomal protein S10p (S20e)	Tetracycline, tigecycline
fig|2508289.5.peg.5113	NTU39_25135	*S12p*	SSU ribosomal protein S12p (S23e)	Streptomycin
fig|2508289.5.peg.2157	NTU39_10845	*rho*	Transcription termination factor Rho	Bicyclomycin
fig|2508289.5.peg.5111	NTU39_25125	*EF-G*	Translation elongation factor G	Fusidic acid
fig|2508289.5.peg.2255	NTU39_11325	*EF-G*	Translation elongation factor G	Fusidic acid

BRC ID	RefSeq locus tag	Gene	Product	Antimicrobial drugs
fig|2508289.5.peg.5146	NTU39_25300	*EF-Tu*	Translation elongation factor Tu	Kirromycin, enacyloxin IIa, pulvomycin
fig|2508289.5.peg.5110	NTU39_25120	*EF-Tu*	Translation elongation factor Tu	Kirromycin, enacyloxin IIa, pulvomycin
fig|2508289.5.peg.5033	NTU39_24745	*MurA*	UDP-N-acetylglucosamine 1-carboxyvinyltransferase (EC 2.5.1.7)	Fosfomycin
fig|2508289.5.peg.4598	NTU39_22600	*MurA*	UDP-N-acetylglucosamine 1-carboxyvinyltransferase (EC 2.5.1.7)	Fosfomycin
fig|2508289.5.peg.2651	NTU39_13235	*BcrC*	Undecaprenyl-diphosphatase BcrC (EC 3.6.1.27), conveys bacitracin resistance	Bacitracin

*Resistance determined by the Bacterial and Viral Bioinformatics Resource Center (BV-BRC) genome annotation pipeline (https://www.bv-brc.org).
BRC, Bioinformatics Resource Center; ID, identification.
